# Gut microbiota remodeling: A promising therapeutic strategy to confront hyperuricemia and gout

**DOI:** 10.3389/fcimb.2022.935723

**Published:** 2022-08-10

**Authors:** Zhilei Wang, Yuchen Li, Wenhao Liao, Ju Huang, Yanping Liu, Zhiyong Li, Jianyuan Tang

**Affiliations:** ^1^ TCM Regulating Metabolic Diseases Key Laboratory of Sichuan Province, Hospital of Chengdu University of Traditional Chinese Medicine, Chengdu, China; ^2^ Hospital of Chengdu University of Traditional Chinese Medicine, Chengdu, China; ^3^ College of Medical Technology, Chengdu University of Traditional Chinese Medicine, Chengdu, China; ^4^ School of Traditional Chinese Medicine, Capital Medical University, Beijing, China

**Keywords:** gout, hyperuricemia, gut microbiota, uric acid, probiotics, prebiotics

## Abstract

The incidence of hyperuricemia (HUA) and gout continuously increases and has become a major public health problem. The gut microbiota, which colonizes the human intestine, has a mutually beneficial and symbiotic relationship with the host and plays a vital role in the host’s metabolism and immune regulation. Structural changes or imbalance in the gut microbiota could cause metabolic disorders and participate in the synthesis of purine-metabolizing enzymes and the release of inflammatory cytokines, which is closely related to the occurrence and development of the metabolic immune disease HUA and gout. The gut microbiota as an entry point to explore the pathogenesis of HUA and gout has become a new research hotspot. This review summarizes the characteristics of the gut microbiota in patients with HUA and gout. Meanwhile, the influence of different dietary structures on the gut microbiota, the effect of the gut microbiota on purine and uric acid metabolism, and the internal relationship between the gut microbiota and metabolic endotoxemia/inflammatory factors are explored. Moreover, the intervention effects of probiotics, prebiotics, and fecal microbial transplantation on HUA and gout are also systematically reviewed to provide a gut flora solution for the prevention and treatment of related diseases.

## 1. Introduction

**Figure 1:**
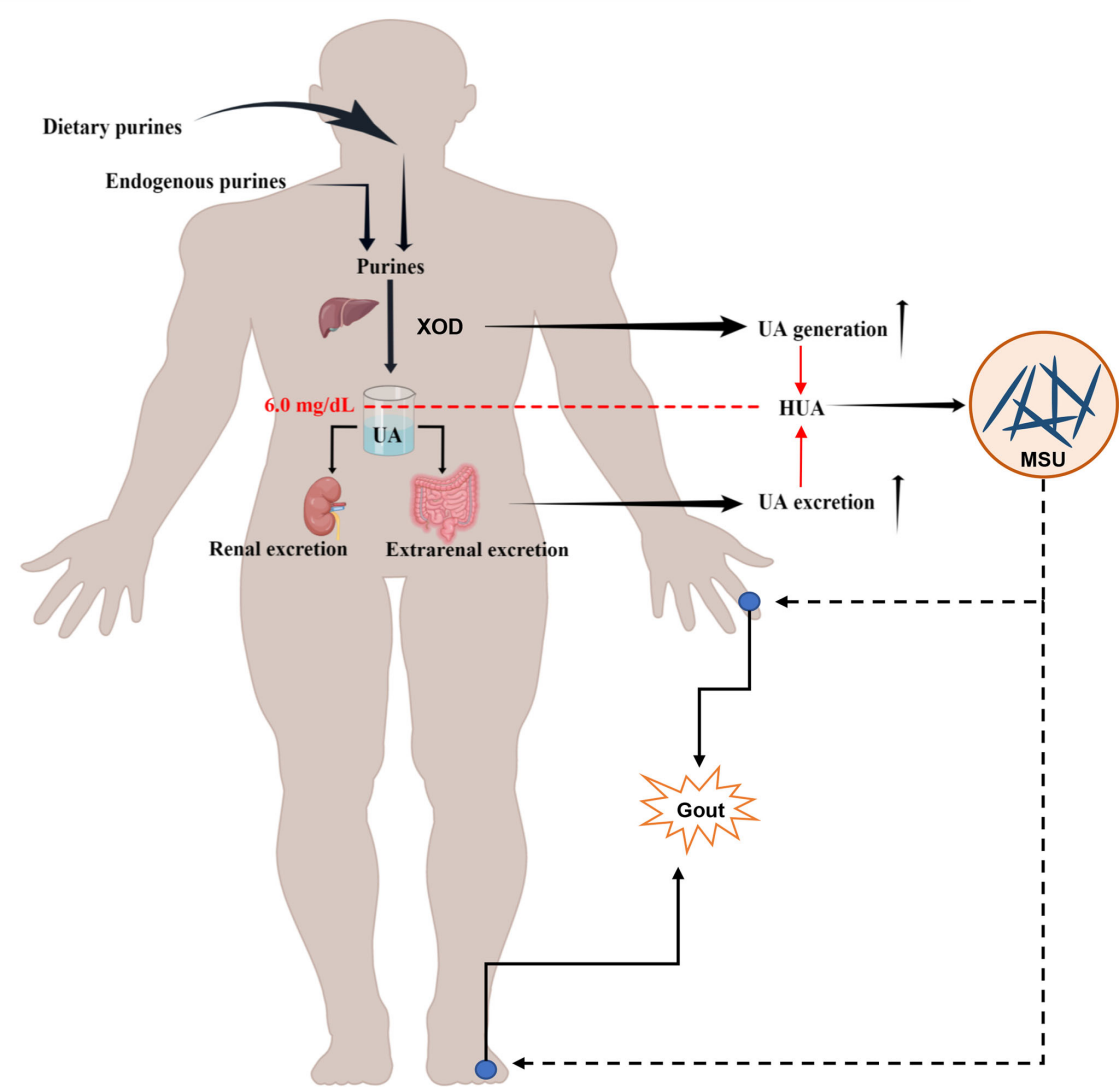
Schematic diagram of the pathophysiological model of HUA and gout formation. HUA (serum UA levels >6.0 mg/dl) is caused by an imbalance of UA metabolism, including an increase in UA production through endogenous purine and exogenous purine metabolism and a decrease in UA excretion owing to the reduction of renal excretion and extrarenal excretion. The deposition of MSU crystals in or around the joints induces gout.

Uric acid (UA) is the terminal metabolite of human purine compounds, and excessive UA production and/or reduced UA excretion can lead to hyperuricemia (HUA, >6 mg/dl) (Bardin and Richette, 2014). Gout is a crystal-related joint disease caused by the deposition of monosodium urate (MSU) crystals in or around the joints. Gout is directly related to HUA caused by disorders of purine metabolism, decreased UA excretion, or increased UA production in the body (**Figure 1**) (Richette and Bardin, 2010; Dalbeth et al., 2016; Dalbeth et al., 2021). In recent years, the incidence of HUA and gout has continued to increase worldwide, which may be related to changes in lifestyle, such as the prevalence of a high-purine diet, fructose beverages, and alcohol consumption (Kolasinski, 2014; Jakše et al., 2019; Yokose et al., 2021). HUA and gout cause increased oxidative stress, endothelial dysfunction, inflammation, platelet adhesion and aggregation, and vasoconstriction (Macfarlane et al., 1983; Krishnan, 2010; Feig, 2014; So and Martinon, 2017; Zhao et al., 2021). HUA and gout are also independent risk factors for cardiovascular disease, metabolic syndrome, and acute kidney injury (Thottam et al., 2017; Bardin and Richette, 2017; Abeles and Pillinger, 2019). The prevalence of HUA and its complications has caused a heavy economic burden on society and families. With the trend of HUA and gout rising and occurring in younger individuals, patients have an urgent need for efficient and safe therapeutic methods or drugs. The scientific understanding of the pathogenesis of related diseases and the selection and optimization of therapeutic drugs have always been the focus of medical research and represent the great opportunities and challenges faced by HUA and gout treatment.

Various microorganisms colonize the human gut and play an irreplaceable role in regulating human energy and metabolism. Structural changes or imbalances in the gut microbiota may cause organism metabolic disorders (Agus et al., 2021; Fan, 2021). Considering the gut microbiota as an entry point to explore the pathogenesis of diseases has become a new hotspot worldwide (Meng et al., 2018; Jackson et al., 2018; Angelucci et al., 2019; Bell et al., 2020), especially in research on investigating the pathogenesis of metabolic-related diseases, such as HUA and gout. Approximately 70% of UA is excreted through the kidneys, and the remainder is mainly excreted with the feces or further catabolized by the gut microbiota. Current studies have found that gut microbiota imbalances exist in patients with HUA and gout (Chu et al., 2021; Yin et al., 2022). In addition, the gut microbiota can be altered after UA reduction (Yu et al., 2018), and probiotics may have UA-lowering effects (Wu et al., 2021; Zhao and Lu, 2022). Therefore, an in-depth study of the relationship between gut microbes and HUA and gout is expected to make it a target for the prevention and treatment of related diseases.

## 2. Characteristics of the gut microbiota in patients with HUA and gout

There are many types of microorganisms in the human intestinal tract. The gut microbiota is composed of three different types of bacteria, namely, beneficial, harmful, and opportunistic pathogenic bacteria; among these, Firmicutes, Bacteroidetes, Actinobacteria, and Proteobacteria are the most important components of the human gut microbiota (Lynch and Pedersen, 2016). In recent years, research has found that the gut microbiota of patients with HUA and gout has also undergone significant changes (**Table 1**).

**Table 1:** Gut microbiota changes in patients with hyperuricemia and gout.

Symptom	Samples	Participants	Excluded indicators	Alterations of the gut microbiota	Refs.
HUA	Feces	A randomly selected sample of the rural residents, aged 50 years or older from a community-based observational study, the Xiangya Osteoarthritis Study	Not applicable	Decrease richness and diversity; alter composition of microbiota; lower relative abundances of genus *Coprococcus*	(Wei and Zhang, 2022)
	Feces	Forty-five patients with asymptomatic HUA and 45 cases in the control group	Heart failure, structural heart disease, and pulmonary heart disease; a history of using antibiotics or probiotics within 3 months; severe liver and kidney dysfunction; abnormal stool morphology	Increase the relative abundances of most abundant flora, including *Faecalibacterium, Gemmiger, Bacteroides, Roseburia, Bifidobacterium*, and *Akkermansia*	(Yang et al., 2021)
	Feces	Asymptomatic HUA patients (*n* = 8), acute gout patients before UA-lowering therapy (*n* = 14), the same acute gout patients after 30 days UA-lowering therapy (*n* = 9), and chronic gout patients after ≥6 months UA-lowering therapy (*n* = 18)	Active systemic infectious diseases; antibiotic treatment within 1 month prior to study enrollment	Exhibit a higher Firmicutes-to-Bacteroidetes (F/B) ratio and a lower *Prevotella*-to-*Bacteroides* (P/B) ratio	(Kim and Yoon, 2022)
Gout	Feces	One hundred two male acute gout patients and 86 age-matched male healthy controls	Age is not within 15–60 years old; antibiotics and glucocorticoid use within 3 months and 1 month, respectively; gastrointestinal diseases, such as gastrointestinal surgery, Crohn’s disease, ulcerative colitis, or acute diarrhea; history of severe, progressive, or uncontrolled cardiac, hepatic, renal, mental, or hematological disease; history of drug abuse	Increase the relative abundances of *Prevotella, Fusobacterium*, and *Bacteroides*; decrease Enterobacteriaceae and butyrate-producing species, such as *Roseburia, Coprococcus*, and *Eubacterium*	(Chu et al., 2021)
	Feces	Twenty-six male patients suffered from gout for at least 12 months without receive any medical treatment within 1 month of study participation, and 26 male volunteers recruited by a routine physical examination	Patients with comorbid disorders; gastrointestinal tract disorders; and receiving antibiotics within 1 month of this study	Upregulate opportunistic pathogens, such as *Bacteroides*, Porphyromonadaceae *Rhodococcus, Erysipelatoclostridium*, and Anaerolineaceae	(Shao et al., 2017)
	Feces	Thirty-five gout patients aged 32–75 years, and 33 healthy individuals aged 28–70 years	Not applicable	*Barnesiella, Parasporobacterium, Paraprevotella, Anaerotruncus, Pseudobutyrivibrio, Bacteroides, Holdemania*, and *Acetanaerobacterium* are enriched	(Guo et al., 2016)
	Feces	Twenty-six healthy participants and 38 patients newly diagnosed with gout without anti-gout drugs, steroids, proton pump inhibitors, nonsteroidal anti-inflammatory drugs, TCM, or any other drugs in 3 months before admission to the study	Participants complicated with metabolic diseases (such as type 2 diabetes, hypertension, and obesity), infective diseases (acute infection in 3 months and chronic infection, such as hepatitis and tuberculosis), tumors, and other systemic diseases	*Fecalibacterium* is the most predominant genus, followed by *Clostridium* and *Cytophaga*	(Lin et al., 2021)
	Feces	Thirty-three gout patients with at least one subcutaneous tophi, 25 gout patients without subcutaneous tophi, and 53 healthy control	Diagnosis of diabetes, chronic renal failure, other rheumatic disease including other crystalline arthropathy (different from MSU crystals), Cushing syndrome, and chronic gastrointestinal diseases. Patients receiving antibiotics, or antiparasitic therapy, or who had diarrhea in the last 3 months	The Proteobacteria phylum and the *Escherichia-Shigella* genus are more abundant	(Méndez-Salazar et al., 2021)

Patients with HUA had reduced microbiota richness and diversity, an altered composition, and a lower relative abundance of *Coprococcus* than the normal controls (Wei and Zhang, 2022). The probiotics *Faecalibacterium*, *Gemmiger*, *Bacteroides*, *Roseburia*, *Bifidobacterium*, and *Akkermansia* were significantly increased in asymptomatic HUA patients compared to the controls (Yang et al., 2021). The increase in probiotics is presumed to be a compensatory phenomenon. Structural changes in the gut microbiota are an important cause of elevated UA levels. Highly metabolically active commensal bacteria, such as *Faecalibacterium*, play an important role in balancing gut immunity as a part of a healthy human microbiome (Miquel et al., 2013; Ferreira-Halder et al., 2017). As one of the first microbial colonizers of the neonatal gut, *Bifidobacterium* plays a key role in physiological development, including the maturation of the immune system and the use of dietary components (Hidalgo-Cantabrana et al., 2017). Through a comparative analysis of the gut microbiota of gout and HUA patients, the gut microbiota diversity in gout patients was lower than that in primary asymptomatic HUA patients (Kim and Yoon, 2022). It is speculated that different gut microbiota in asymptomatic HUA patients may prevent gout development, while differences in gout patients perhaps play a role in gout induction. Furthermore, an asymptomatic HUA group showed a higher Firmicutes-to-Bacteroidetes (F/B) ratio and a lower *Prevotella*-to-*Bacteroides* ratio (P/B) (Kim and Yoon, 2022). The F/B ratio is widely believed to have an important impact on the maintenance of normal intestinal homeostasis (Stojanov and Berlec, 2020). An increase or decrease in the F/B ratio is a manifestation of an organism’s dysbiosis.

The metagenomes of gout patients significantly differed from those of healthy controls, and the richness and diversity of the gut microbiota were reduced in gout patients (Shao et al., 2017; Chu et al., 2021). The relative abundances of *Prevotella*, *Fusobacterium*, and *Bacteroides* were increased in gout patients, while the relative abundances of Enterobacteriaceae and butyrate-producing species were decreased (Chu et al., 2021). Meanwhile, studies have also shown the upregulation of opportunistic pathogens, such as *Bacteroides*, *Rhodococcus*, *Erysipelatoclostridium*, and Anaerolineaceae, in gout patients (Shao et al., 2017). The gut microbes of gout patients were rich in *Bacteroides caccae*, *Bacteroides xylanisolvens*, *Bacteroides ovatus*, and *Eubacterium rectale*, but relatively deficient in *Faecalibacterium prausnitzii* and *Bifidobacterium pseudocatenulatum (* Guo et al., 2016). However, contrary findings showed that *Fecalibacterium* was the most dominant genus detected in untreated gout patients, followed by *Clostridium* and *Cytophaga (* Lin et al., 2021). An analysis of the gut microbiota in gout patients with and without tophi revealed that the genera *Phascolarctobacterium*, *Bacteroides*, *Akkermansia*, and *Ruminococcus*_gnavus_group had the lowest species diversity but a higher abundance in gout patients without tophi (Méndez-Salazar et al., 2021). Proteobacteria and *Escherichia coli*-*Shigella* were more abundant in tophi patients than controls (Méndez-Salazar et al., 2021). The differences of the gut microbiota in different studies are believed to be caused by the following reasons. On the one hand, differences may exist in purine metabolism, UA metabolism, and amino acid metabolism between HUA and gout patients, resulting in the transformation of gut microbiota. On the other hand, differences in sex and age affect the structure and composition of the gut microbiota (Kim and Jazwinski, 2018; Aron-Wisnewsky et al., 2020). In addition, although most clinical studies have ruled out the influence of underlying diseases and antibiotic treatment, other factors, such as diet, smoking, and alcohol consumption, are important factors in regulating changes in the gut microbiota (Engen et al., 2015; Huang and Shi, 2019; Zmora et al., 2019). In summary, alterations in the gut microbiota are clearly closely related to various factors. Therefore, an analysis of the gut microbiota in specific populations of HUA and gout patients could help improve the understanding of gut microbiota therapy.

## 3. The gut microbiota in the pathogenesis of HUA and gout

### 3.1 Diet and intestinal microbiota homeostasis

**Figure 2:**
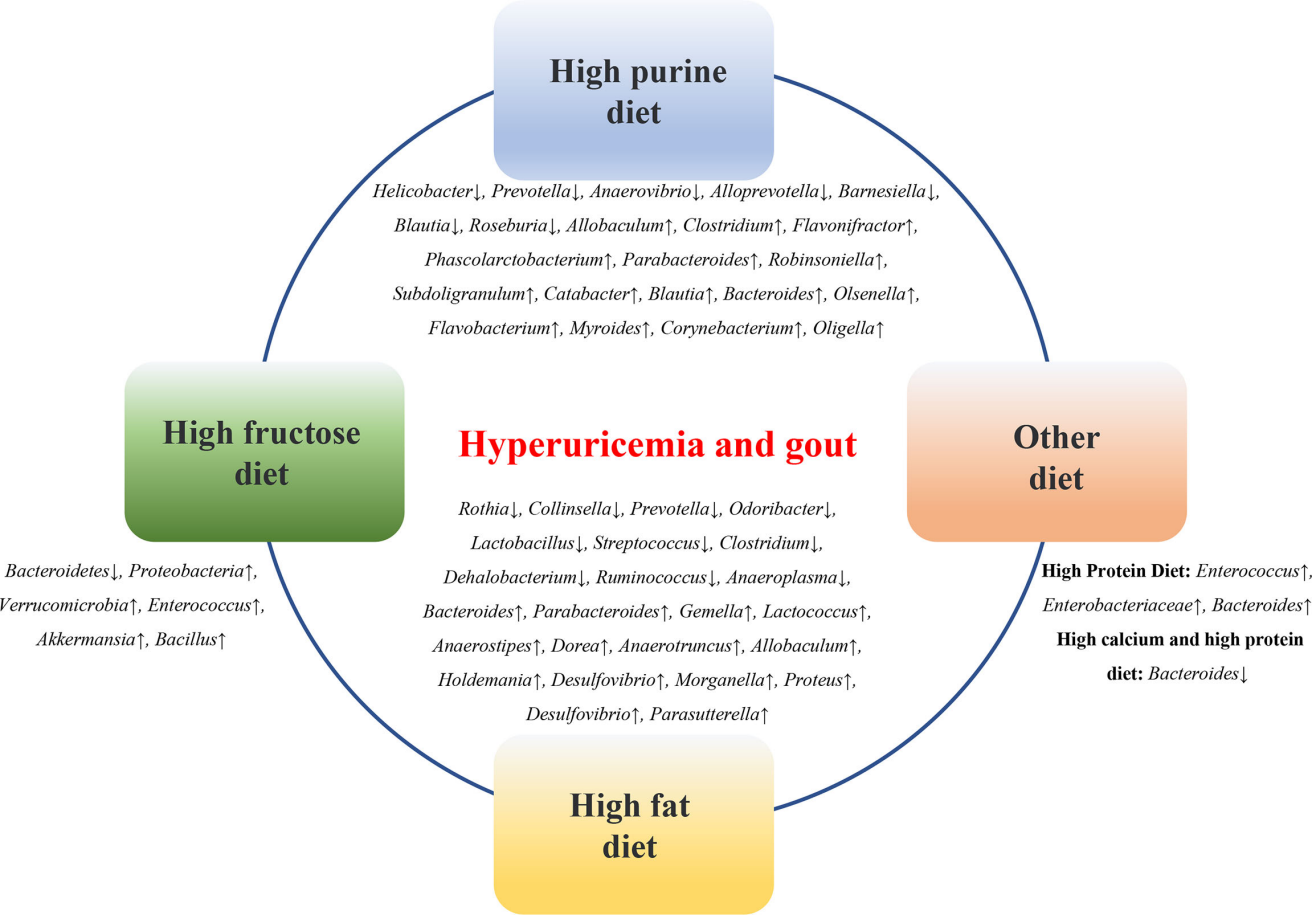
Diets affect the microbial composition of the gastrointestinal tract. High-fructose diet, high-fat diet, high-purine diet, etc. can lead to changes in the composition of intestinal flora in animal models of HUA and gout.

Diet is an important factor affecting the microbial composition of the gastrointestinal tract. A high-fructose diet, high-fat diet, high-purine diet, and high-oxalate diet can lead to changes in the composition of the gut microbiota in animal models of HUA and gout (**Figure 2**).

#### 3.1.1 High-purine diet

Eighty percent of UA is derived from the degradation of endogenous purines, and 20% of UA is derived from exogenous purines, such as food. A high-purine diet, such as seafood, animal offal, and alcohol consumption, is a risk factor for HUA and gout and an important cause of gut microbiota imbalance in patients. Compared with the control group, the relative abundances of Firmicutes and Actinobacteria were significantly increased, and the relative abundances of Bacteroidetes and Proteobacteria were significantly reduced in a purine diet-induced HUA mouse model (Wan et al., 2020). In a quail HUA model induced by a high-purine diet, the proportion of Prevotellaceae was significantly higher than that in the normal group, while the abundance of *Helicobacter* was low (Bian et al., 2020). In a yeast and purine diet-induced HUA rat model, the diversity and abundance of the gut microbiota were altered, and the relative abundances of *Prevotella*, *Anaerovibrio*, *Alloprevotella*, and *Barnesiella* at the genus level were lower than those in the control rats, while *Allobaculum*, *Clostridium*_XlVa, *Flavonifractor*, *Phascolarctobacterium*, *Clostridium*_XVIII, *Parabacteroides*, *Robinsoniella*, *Subdoligranulum*, *Catabacter*, *Blautia*, *Bacteroides*, and *Olsenella* were relatively more abundant than those in the controls (Liu et al., 2020). In a purine diet-induced hyperuricemic nephropathy rat model, *Flavobacterium*, *Myroides*, *Corynebacterium*, *Alcaligenaceae*, *Oligella*, and other opportunistic pathogens were significantly increased, while the short-chain fatty acid (SCFA)-producing bacteria *Blautia* and *Roseburia* were significantly decreased (Pan et al., 2020). The changes in these microbial groups suggest that transformation in gut microbes may play a role in HUA and gout.

#### 3.1.2 High-fructose diet

The risk of obesity, diabetes, cardiovascular disease, and metabolic syndrome is linked to the consumption of beverages containing sugar or high fructose corn syrup (Tappy and Lê, 2010; Bray, 2013; Jensen et al., 2018). Excessive fructose intake is an important reason for the increased prevalence of gout and HUA (Hak and Choi, 2008; Li et al., 2018; Nakagawa et al., 2019). Fructose metabolism activates adenosine monophosphate deaminase, promoting purine degradation and inosine production and resulting in elevated serum UA (Thottam et al., 2017). Research has shown that a large amount of fructose can activate nicotinamide adenine dinucleotide phosphate oxidase, which further prevents the excretion of UA through the ileum, resulting in an increase in the serum UA levels in the human body (Kaneko et al., 2017). A high-fructose diet can significantly reduce the diversity of gut microbes in the short term, with a significant increase in the ratio of Firmicutes to Bacteroidetes and a decrease in the proportion of probiotics such as *Lactobacillus (* Silva et al., 2018). The changes in the gut microbiota further reduce butyric acid and glutamate in the intestinal tract and increase the production of fructose, succinic acid, taurine, tyrosine, and xylose, leading to a disturbance in the intestinal microecology and further impairing the intestinal barrier function (Silva et al., 2018). Studies have found the following two main types of flora in mice after high-fructose feeding: increases in Proteobacteria, and significant decreases in *Bacteroidetes (* Do et al., 2018). In a mouse model of HUA induced by high fructose, the abundance of Bacteroidetes was significantly decreased, and the abundance of Verrucomicrobia, *Enterococcus*, *Akkermansia*, and *Bacillus* was increased (Wang et al., 2019). In addition, a high consumption of fructose can promote intestinal malnutrition and lipopolysaccharide (LPS)-related inflammation while accelerating the degradation of purines (Wang and Qi, 2020). Therefore, lowering the intake of fructose and adopting a healthier diet may help better manage HUA and gout and reduce related diseases. Meanwhile, further observations and evaluations of the actual efficacy of specific dietary measures in HUA and gout patients in clinical practice are needed.

#### 3.1.3 High-fat diet

As a public health problem, a high-fat diet has been shown to be associated with various digestive system diseases, cardiovascular diseases, urinary system diseases, tumors, etc. and can accelerate the occurrence of diseases due to inflammation and metabolic changes (Yin et al., 2018; Tong et al., 2021). A high-fat diet is closely associated with changes in the gut microbiota. Studies have shown that at the genus level, 12 genera (*Bacteroides*, *Parabacteroides*, *Gemella*, *Lactococcus*, *Anaerostipes*, *Dorea*, *Anaerotruncus*, *Allobaculum*, *Holdemania*, *Desulfovibrio*, *Morganella*, and *Proteus*) were enriched in HUA rats induced by a high-fat diet containing 10% yeast extract, but 10 other genera (*Rothia*, *Collinsella*, *Prevotella*, *Odoribacter*, *Lactobacillus*, *Streptococcus*, *Clostridium*, *Dehalobacterium*, *Ruminococcus*, and *Anaeroplasma*) were less abundant (Yu et al., 2018). Another study showed that the relative abundances of Muribaculaceae, *Bacteroides*, and Lachnospiraceae were significantly decreased in a gout model induced by MSU crystals combined with high-fat diet feeding compared with the normal control group (Feng et al., 2022). In a gout model induced by a combination of MSU crystals and high-fat diet feeding, changes in the gut microbiota were induced, including increased levels of *Desulfovibrio* and *Parabacteria (* Lin et al., 2020).

#### 3.1.4 Other diets

Studies have found that the expression of adenosine triphosphate-binding cassette superfamily G member 2 (ABCG2) was significantly increased in an oxonic acid diet-induced HUA rat model, supplementation with probiotics could reduce this expression, and significant differences in the microbiota were observed between the treated and untreated fecal samples (García-Arroyo et al., 2018). *Enterococcus*, Enterobacteriaceae, and Bacteroidetes were enriched in the cecum of high-protein diet-induced gout goslings (Xi et al., 2020). A high-calcium and high-protein diet-induced gout model exhibited liver and kidney damage in geese, an impaired intestinal barrier, and a significantly decreased abundance of *Bacteroides* (Ma et al., 2021). Implementing dietary solutions for the closely linked diet-gut microbiota-HUA/gout is a new challenge and opportunity, which is of great significance for the prevention and treatment of HUA and gout.

### 3.2 Gut microbiota and metabolism of purine, UA, and amino acids

#### 3.2.1 Gut microbiota and purine metabolism

**Figure 3:**
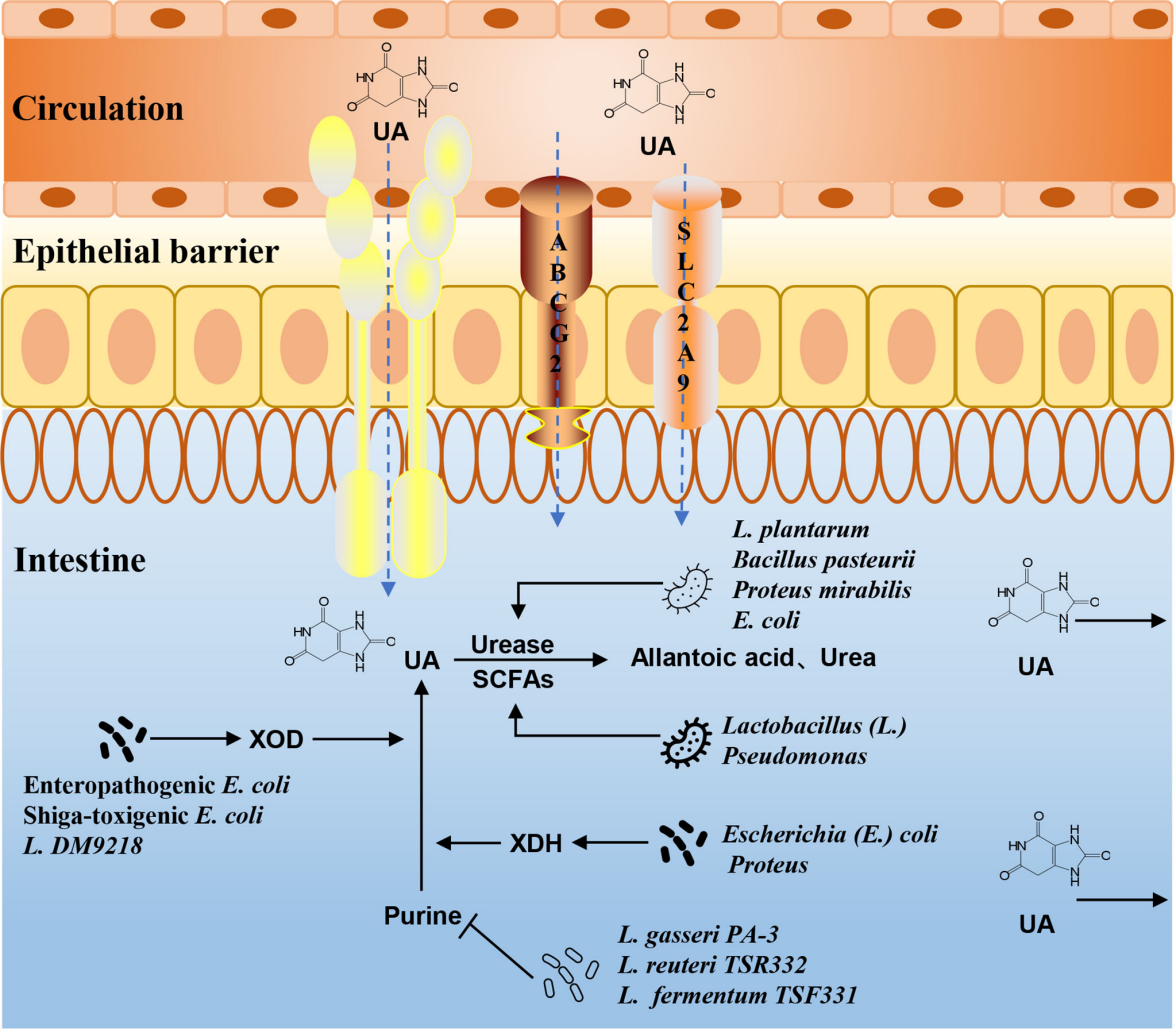
The mechanism of gut microbiota in UA and purine metabolism. The intestinal flora can affect purine metabolism, XOD, XDH, and urease activities, or the composition of SCFAs, and then alter the production and excretion of UA.

UA is the final product of purine metabolism in the human body. Purine nucleotides are hydrolyzed into adenine and guanine, deaminated to form xanthine, and then oxidized to form UA. The gut microbiota can affect the metabolism of purine. *Lactobacillus gasseri* PA-3 in the intestine can absorb and utilize purine, thereby reducing the intestinal absorption of purine in the diet and reducing the serum UA levels (Yamada et al., 2016). Xanthine dehydrogenase (XDH), which is responsible for purine oxidative metabolism, can be secreted by bacteria of the genus *Escherichia*, such as *E. coli (* Wang et al., 2017). *Lactobacillus* DM9218 can effectively reduce the serum UA levels in HUA rats by inhibiting xanthine oxidase (XOD) activity (Li et al., 2014). Shiga-toxigenic *E. coli* (STEC) and enteropathogenic *E. coli* (EPEC) can promote the release of XOD in intestinal epithelial cells, accelerate the decomposition of hypoxanthine and xanthine, and convert more purines for UA (Crane et al., 2013). *Lactobacillus reuteri* TSR332 and *Lactobacillus fermentum* TSF331 can control the development of HUA by degrading purine (Kuo et al., 2021). Intestinal microbes play an important role in the process of purine metabolism (**Figure 3**). How to use these beneficial intestinal bacteria to regulate purine metabolism and then affect the production of UA is a problem that should be considered in current research.

#### 3.2.2 Gut microbiota and UA metabolism

The gut is a main pathway of UA excretion, and the microbial environment in the gut is the basis for this function. There are two main ways to excrete UA in healthy people. Two-thirds are excreted from the urethra through renal tubular secretion, and the remaining one-third is excreted through the intestines. UA transporters in intestinal epithelial cells are responsible for transporting UA from the blood to the intestinal lumen, where it is directly excreted or broken down by the gut microbiota. *Lactobacillus* and *Pseudomonas* promote the decomposition and excretion of UA in the intestine by producing SCFAs (Wrigley et al., 2020). The gut microbiota can also regulate intestinal epithelial ABCG2, urate transporter soluble carrier protein 2 family member 9 (SLC2A9), and other UA transporters, which, in turn, affect UA metabolism (Hosomi et al., 2012; DeBosch et al., 2014). The activities of UA metabolism-related enzymes are also closely related to the gut microbiota. Uricase, which converts UA into allantoin and urea, is widely found in *Bacillus pasteurii*, *Proteus mirabilis*, *E. coli*, etc. (Christians and Kaltwasser, 1986; Rando et al., 1990; Nakagawa et al., 1996). *Lactobacillus* sp. OL-5, *Lactobacillus plantarum* Mut-7, and *L. plantarum* Dad-13 had higher intracellular uricase activity (Handayani and Hidayat, 0000). Experimental studies have shown that the abundance of *Lactobacillus*, *Streptococcus*, and *Clostridium* with purine absorption and UA decomposition decreased in the gut microbiota of HUA rats, while the abundance of *Proteus*, which secretes XDH, increased (Yu et al., 2018). The changes in the microbiota may play an important role in the regulation and metabolism of the UA levels (**Figure 3**).

#### 3.2.3 Gut microbiota and amino acid metabolism

Amino acid metabolism also plays an important role in the development of HUA and gout (Mahbub et al., 2017; Pan et al., 2020; Huang et al., 2020). A reduced species abundance of Enterobacteriaceae was associated with amino acid metabolism and environmental perception, which together resulted in increased serum UA and C-reactive protein levels in gout patients (Chu et al., 2021). After metagenomics and cluster analysis of feces from gout patients and healthy people, the samples were divided into high gout clusters dominated by *Bacteroides* and low gout clusters with increased *Faecalibacterium (* Henson, 2021). The high gout cluster exhibited increased synthesis of the amino acids D-alanine and L-alanine and by-products of branched-chain amino acid catabolism, while the low gout cluster exhibited increased the production of butyrate, the sulfur-containing amino acids L-cysteine and L-methionine, and the L-cysteine catabolite H_2_S (Henson, 2021). Using a urate oxidase (Uox)-KO mouse model that spontaneously developed overt HUA and urate nephropathy, the gut microbiota composition and function were altered, and a distinct metabolome existed. Among them, amino acid metabolism plays a key role, and characteristic metabolites are strongly influenced by differential bacterial genera. Furthermore, an impaired gut integrity and profound alterations in the solute carrier family lead to dysregulated amino acid transport, which, in turn, affects the serum UA levels and CD4+ Th17-driven inflammation (Song et al., 2022). Of course, research investigating the effect of gut microbiota on amino acid metabolism needs to be further conducted.

In summary, the current research mainly focuses on the influence of the gut microbiota on purine metabolism, UA metabolism, and amino acid metabolism. Whether these metabolic processes can affect changes in the gut microbiota and thereby alter HUA and gout symptoms has not been discussed. Therefore, the next step should be to further explore the interaction between the gut microbiota and these metabolic processes to provide a theoretical research basis for the treatment of HUA and gout.

### 3.3 Gut microbiota and the secretion of LPS

**Figure 4:**
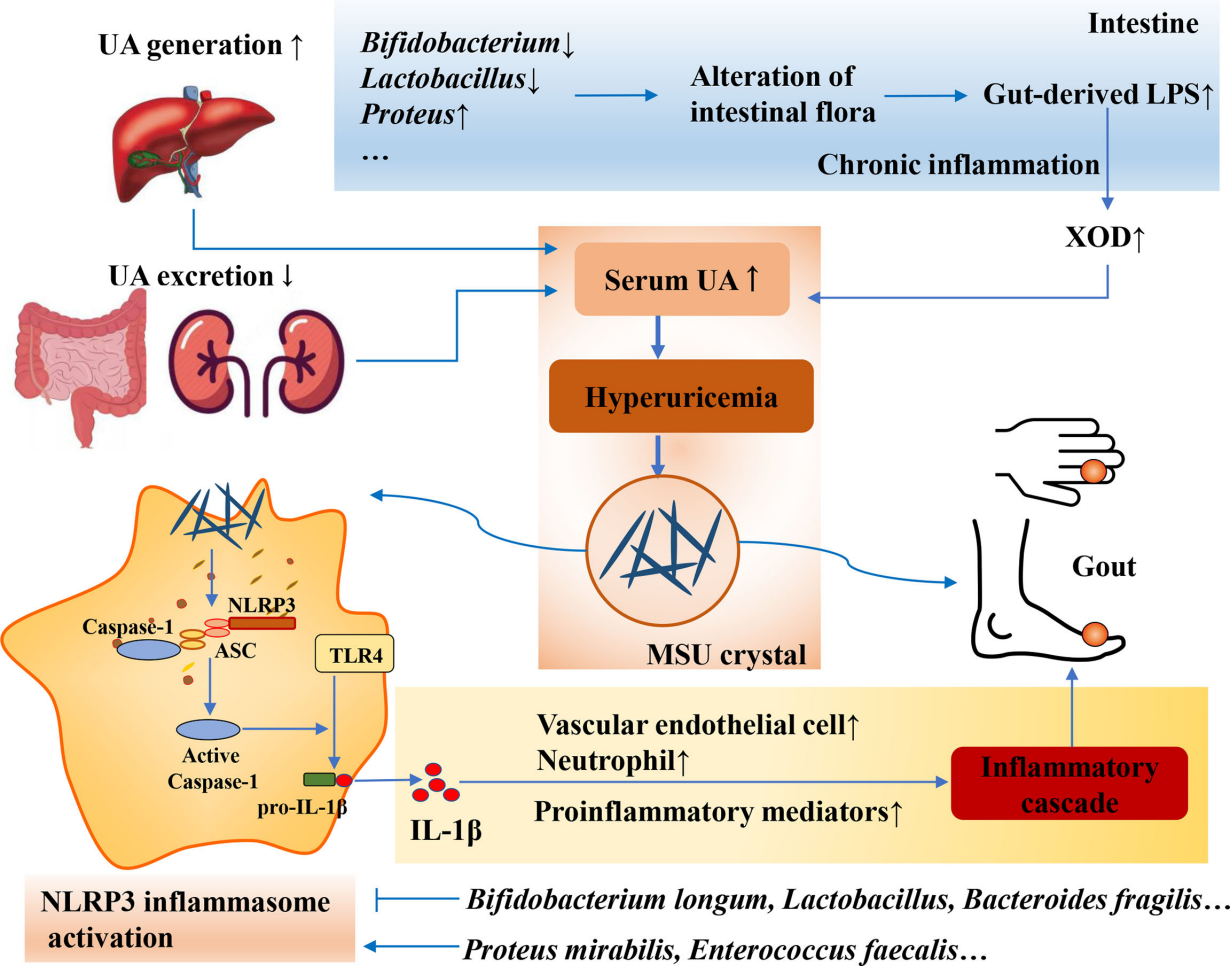
Intestinal flora affects the secretion of LPS and inflammatory response. Loss of gut barrier resulting from gut dysbiosis leads to gut-derived LPS translocation and chronic inflammation, resulting in the increase of SOD activity and serum UA. Moreover, gut microbiota can also modulate NLRP3 inflammasome activation, and the commensal microbiota shapes the ability of the host to respond to acute inflammatory stimuli that are dependent on the extra-intestinal inflammasome.

LPS is a component of the cytoderm of Gram-negative bacteria in the gut microbiota. Disturbances in the intestinal microenvironment can significantly suppress the activity of Gram-negative bacteria. Furthermore, the increased secretion of LPS can prompt the body to produce a large amount of cytokines, increase the permeability of the intestinal wall, and induce low-grade chronic inflammation, which is called “metabolic endotoxemia” (Fuke and Nagata, 2019; Ota et al., 2020). Recent studies have found that LPS is closely related to metabolic syndrome, obesity, and other diseases (Ghosh et al., 0000; Miller et al., 2005; Qin et al., 2007; Hersoug et al., 2018). The loss of the gut barrier resulting from gut dysbiosis leads to gut-derived LPS translocation, which plays an important role in the development of goose gout by interfering with renal function (Xi et al., 2019). Changes in the gut microbiota can increase intestinal permeability, leading to changes in LPS entering the circulatory system, thereby controlling metabolic endotoxemia, inflammation, and related diseases (Cani et al., 2008). XOD is a key enzyme in the production of UA, and an increase in XOD activity leads to an increase in UA production. Studies have shown that chronic inflammation caused by elevated serum LPS levels is often accompanied by elevated XOD activity (Ramos et al., 2018). The levels of inflammatory cytokines, LPS, and XOD activity in Uox-KO mice were significantly higher than those in wild-type mice (Guo et al., 2021). The abundance of *Bifidobacterium* and *Lactobacillus* in feces from HUA mice was significantly reduced, and the levels of UA, XOD activity, and LPS were significantly increased compared with those in normal mice (Cao et al., 2017). After a certain period of probiotic treatment, the number of *Bifidobacterium* and *Lactobacillus* in the gut microbiota increased, and the activities of serum UA, LPS, and XOD decreased (Cao et al., 2017). The above studies suggest that the mechanism of HUA may be related to LPS-activated XOD activity caused by a gut microbiota imbalance (**Figure 4**).

### 3.4 Gut microbiota and inflammatory response

The gut microbiome and SCFAs play critical roles in regulating the body’s inflammatory response to MSU crystals. SCFAs, including acetate, butyrate, and propionate, are the main metabolites of dietary fiber fermented by gut microbes (Sivaprakasam et al., 2016). It is also the key to regulating dietary fiber and the intestinal microbiota, and has important physiological functions, such as reducing the intestinal inflammatory response, improving intestinal epithelial barrier function, and maintaining fluid balance and energy metabolism (Tan et al., 2014). Studies have shown that the inflammatory response to an intra-articular injection of MSU crystals is dependent on SCFAs produced by microbes in the gut and that adding acetate to the drinking water of germ-free mice can restore this inflammatory response (Vieira et al., 2015). After comparing the differences in the gut microbiome and serum SCFAs in 20 patients with acute gouty arthritis between acute and convalescent states, the results showed that the gut bacterial composition significantly changed and that the acetate levels were significantly elevated in a convalescent state (Park and Lee, 2022). In addition, a study showed that several metabolites in feces from gout patients that play a role in regulating inflammation differ from those in normal people, such as increased succinic acid (Shao et al., 2017).

Inflammasome activation can be induced by various microbial pathogens, often mediating host defense by activating inflammatory responses and limiting pathogen replication. Among them, the nod-like receptor family pyrin domain containing 3 (NLRP3) inflammasome is involved in the development of gout as a sensor of metabolic stress (Martinon et al., 2006; So and Martinon, 2017; Jhang et al., 2018). In addition to its role in defense against pathogens, recent studies have shown that the gut microbiota can modulate NLRP3 inflammasome activation (**Figure 4**). In the absence of microbiota, the production of SCFAs, which are required for inflammasome assembly and interleukin (IL)-1β production, is reduced, and macrophages from germ-free animals produce limited oxygen and inflammasome assembly (Vieira et al., 2015). These results clearly demonstrate that the commensal microbiota shapes the ability of the host to respond to acute inflammatory stimuli that are dependent on the extraintestinal inflammasome. Enterobacteriaceae, especially the pathogen *Proteus mirabilis*, induce the activation of NLRP3 inflammasome (Seo et al., 2015). *Enterococcus faecalis* activates the NLRP3 inflammasome, leading to the increased secretion of IL-1β and the generation of pyroptosis (Ran et al., 2019; Yin et al., 2020). *Lactobacillus* reduces reactive oxygen species production by restoring the abnormal mitochondrial membrane potential, thereby inhibiting the activation of NLRP3 (Saber and Abd El-Fattah, 2021). *Bifidobacterium longum* may downregulate the expression of IL-18 and IL-1β by inhibiting the NLRP3 inflammasome (Gu et al., 2016). *Bacteroides fragilis* negatively regulates the NLRP3-mediated inflammatory signaling pathway, inhibits the activation of macrophages and the secretion of proinflammatory mediators, such as IL-18 and IL-1β, and reduces the level of intestinal inflammation (Shao et al., 2021). Currently, the regulation of these florae is mainly concentrated in diseases such as enteritis, and the therapeutic effect on HUA and gout needs to be further developed and utilized. Probiotic intervention is promising for modulating the NLRP3 inflammasome signaling pathway to improve HUA and gout.

## 4. Gut microbiota as a potential therapeutic target in HUA and gout

### 4.1 Probiotics

Currently, the main drugs used to lower UA are XOD inhibitors and uricosuric drugs, but these drugs have certain side effects. Relevant progress has been achieved in the use of probiotics for the treatment of HUA and gout. *Lactobacillus*, *Bifidobacterium*, and *Saccharomyces* have a long history of safe and effective use as probiotics (Sanders et al., 2019).

Regarding probiotic treatment, a study found that *L. gasseri* PA-3 can improve the UA levels in healthy people, HUA patients, and gout patients (Yamanaka et al., 2019). Experiments in an *in vitro* colon model found that *L. gasseri* PA3 decreased gut microbiota diversity, increased the relative abundance of *Lactobacillus* and *Escherichia*, and decreased the relative abundance of *Bacteroides* and *Phascolarctobacterium (* Xiang et al., 2019). *L. rhamnosus* R31, *L. rhamnosus* R28-1, and *L. reuteri* L20M3 promote the production of SCFAs in a purine-independent manner and alleviate the serum and urine UA concentrations in HUA mice (Ni et al., 2021). These strains also reversed the elevated LPS concentrations, liver inflammation, and kidney damage associated with HUA (Ni et al., 2021). The serum UA levels in mice fed Limosilactobacillus fermentum JL-3 were lower than those in the control group (Wu et al., 2021). The JL-3 strain also restored some inflammatory markers and oxidative stress indicators [IL-1β, malonaldehyde, creatinine (CRE), and blood urea nitrogen (BUN)] associated with HUA, while gut microbial diversity results showed that JL-3 can regulate the gut microbiota imbalance caused by HUA (Wu et al., 2021). *Lactobacillus brevis* DM9218 reduces the serum UA levels and hepatic XOD activity in fructose-fed mice (Wang et al., 2019). It can prevent liver damage caused by high fructose and delay the accumulation of UA by degrading inosine, regulating intestinal dysbiosis, enhancing intestinal barrier function, and reducing hepatic LPS (Wang et al., 2019). In a model of HUA induced by potassium oxonate and a high-purine diet, *L. reuteri* TSR332 and *L. fermentum* TSF331 stabilized the serum UA levels in rats, and no obvious side effects were observed (Kuo et al., 2021). There are many types of probiotics, and their mechanisms of action and their ability to colonize the gut are different. Therefore, rigorous and reliable data are still needed to further prove the effect of probiotics on HUA and gout.

### 4.2 Prebiotics

Published in 2017, prebiotics are defined as “substrates selectively utilized by host microorganisms with health benefits”, which expands the concept of prebiotics to potentially include noncarbohydrate substances, applications to body parts other than the gastrointestinal tract, and many categories other than food (Gibson et al., 2017). Prebiotics can play a role in the treatment of HUA and gout by altering the structure of the gut microbiota (**Table 2**).

**Table 2:** Experiment and mechanism of prebiotics in prevention of HUA and gout targeting gut microbiota.

Categories	Compounds/extracts	Dose and methods	Study type and model	Alterations of the gut microbiota	Mode of action/mechanism	Refs.
Anti-gout drugs	Febuxostat	Febuxostat tablets for 3 months	Thirty-eight patients diagnosed with gout according to the diagnostic criteria	*Lachnospiraceae Clostridium, Fecalibacterium, Cytophaga, Dorea, Roseburia, Ruminococcaceae Clostridium, Clostridiaceae Clostridium, Alistipes, Succinispira, Sporobacter, Campylobacter, Lachnospira, Robinsoniella, Lactonifactor, Butyrivibrio, Rothia, Pseudomonas*, and *Pediococcus*↑; *Millisia, Leifsonia, Paracoccus*, and *Eggerthella*↓	Biochemical metabolic indexes involved in liver and kidney metabolism are significantly associated with the gut microbiota composition	(Lin et al., 2021)
	Allopurinol	The administration of allopurinol (9 mg/kg) was initiated on the third week and continued for another 6 weeks	A rat model of HUA induced by high-fat feed containing 10% yeast extract	*Bifidobacterium* and *Collinsella*↑; *Adlercreutzia, Anaerostipes, Bilophila, Morganella*, and *Desulfovibrio*↓	Renovate the disorder of nucleotide metabolism in the gut microbiota	(Yu et al., 2018)
	Benzbromarone	The administration of benzbromarone (9 mg/kg) was initiated on the third week and continued for another 6 weeks	A rat model of HUA induced by high-fat feed containing 10% yeast extract	*Bifidobacterium, Collinsella*, and *Proteus*↑; *Adlercreutzia, Anaerostipes, Butyricimonas*, and *Ruminococcus*↓	Renovate the disorder of lipid metabolism in the gut microbiota	(Yu et al., 2018)
TCM formulas	A decoction of a TCM formula CoTOL (1.82 g/ml)	CoTOL (0.4 ml/20 g) was administrated by gavage on the fifth week, once a day for 4 weeks	A mouse model of obese HUA inoculated with XOD-producing bacteria, *Streptococcus faecalis*	*Akkermansia*↑; *Bacteroides* and *Alloprevotella*↓	Regulate material metabolism; improve the structure or function of intestinal flora	(Gao et al., 2020)
	Simiao decoction (0.2, 0.4, and 0.8 g/ml)	Oral gavage with 4.0, 8.0, and 16.0 g/kg Simiao decoction for 29 days	Gouty arthritis mouse model induced by high-fat diet (10% yeast extract) and MSU crystals (25 mg/ml in PBS/mouse per 10 days)	*Prevotella*, *Escherichia-Shigella*, *Klebsiella*, *Megamonas*, *Enterococcus*, *Phascolarctobacterium*, Prevotellaceae Nk3b31, and Ruminococcaceae UCG-014↓	Suppress NLRP3 inflammasome expression; reduce gut apoptosis, affect lipid metabolism, and restore gut microbiota *via* reducing potential pathogens	(Lin et al., 2020)
	A decoction of *Cichorium intybus* L. (chicory)	Chicory inulin water solution (6.6, 13.3, and 16.7 g/kg) by intragastric administration for 60 days	A quail model of HUA induced by yeast extract powder	*Bifidobacterium*, and Erysipelotrichaceae↑; Helicobacteraceae↓	Modulate the imbalance of gut microbiota; suppress LPS/TLR4 axis inflammatory reaction; increase UA excretion by intestines; enhance the mRNA and protein expressions of ABCG2	(Wang et al., 2017; Bian et al., 2020)
Polysaccharides	Polysaccharide from *Ulva lactuca* (ULP)	ULP (300 mg/kg) for 2 weeks	A mouse model of HUA induced by hypoxanthine (300 mg/kg) and oteracil potassium (250 mg/kg)	*Dubosiella, Lactobacillus, Mucispirillum, Parasutterella, Weissella, Kurthia*, and *Bifidobacterium*↑; *Staphylococcus, Escherichia−Shigella, Alloprevotella*, Lachnospiraceae_NK4A136_group, and *Ruminococcus*↓	Decrease the levels of serum UA, BUN, and CRE; inhibit serum and hepatic XOD activities; improve renal injury; elevate the helpful microbial abundance, and decline the harmful bacterial abundance; restore the gut microbiome homeostasis	(Li et al., 2021)
	*Enteromorpha prolifera* polysaccharide (EPP)	EPP (300 mg/kg) for 2 weeks	A mouse model of HUA induced by hypoxanthine (300 mg/kg) and oteracil potassium (250 mg/kg)	*Alistipes* and *Parasutterella*↑; *Firmicutes/Bacteroidetes (F/B)* ratio↓	Reduce serum UA, BUN, XOD, and hepatic XOD; upregulate UA excretion genes ABCG2, OAT1, and NPT1 and downregulate UA resorption gene URAT1; maintain the stability of the intestinal flora	(Li et al., 2021)
	Inulin	Inulin by oral gavage (9.5 g/kg) for 7 weeks	A mouse model of HUA induced by urate oxidase (Uox)-knockout	SCFAs-producing bacteria (e.g., *Akkermansia* and *Ruminococcus*)↑	Increase the expressions of ABCG2 in intestine; downregulate expression and activity of hepatic XOD; repair the intestinal epithelial barrier as evidenced by increased levels of intestinal tight junction proteins (ZO-1 and occluding)	(Guo et al., 2021)
	β-carotene and green tea powder	Supplemented with 0.05% β-carotene and 2% green tea powder in the high-fat diet	A mouse model of gouty arthritis was induced by high-fat diet (10% yeast extract) and MSU crystals (1 mg mixed in 40 μl of PBS) once every 10 days for 6 weeks	Muribaculaceae, Ruminococcaceae_UCG-014, Lachnospiraceae_NK4A136_group, and *Bacteroides*↑	Reduce the levels of serum UA and pro-inflammatory cytokines IL-1β, IL-6, and TNF-α; improve the gut microbiota profile; and reduce the metabolic levels of purines and pyrimidines	(Feng et al., 2022)
Phenols	The ethyl acetate extract of *Camellia japonica* bee pollen	The ethyl acetate extract of *Camellia japonica* bee pollen (2 and 4 g/kg) was administrated by gavage for 3 weeks	Potassium oxonate-induced HUA mouse model	*Lactobacillus, Allobaculum Adlercreutzia*, and *Clostridia*↑	Suppress TLR4/MyD88/NF-κB signaling pathway and NLRP3 inflammasome activation; modulate gut microbiota structure	(Xu et al., 2021)
	Chlorogenic acid (CGA)	CGA (30 and 60 mg/kg) was orally administered to the mice for 19 days	A mouse model of HUA induced by hypoxanthine (300 mg/kg) and potassium oxonate (300 mg/kg)	*Bacteroides*, Prevotellaceae UGC-001, and *Butyricimonas*↑	Inhibit the activation of the TLR4/MyD88/NF-κB signaling pathway; increase the relative abundance of SCFA-producing bacteria; reverse the purine metabolism and glutamate metabolism functions of gut microbiota	(Zhou et al., 2021)
Peptides	Tuna meat oligopeptides (TMOP)	50 and 300 mg/kg TMOP by gavage for 8 weeks	A mouse model of HUA induced by purine-rich solution (containing 200 mg/kg hypoxanthine and 30 mg/kg yeast extract) and 250 mg/kg potassium oxonate in combination by oral gavage once a day for 8 weeks	*Bacteroidia*, *Bacilli*, *Actinobacteria*, and *Epsilonproteobacteria*↑	Alleviate HUA and renal inflammation phenotypes; reprogram UA metabolism pathways; inhibit the activation of NLRP3 inflammasome and TLR4/MyD88/NF-κB signaling pathways; repair the intestinal epithelial barrier; reverse the gut microbiota dysbiosis and increase the production of SCFAs	(Han et al., 2020)
	Two novel hexapeptides (GPAGPR and GPSGRP) from Apostichopus japonicus hydrolysate	200 μl of GPAGPR or GPSGRP (10 mg/kg) by oral gavage for 12 weeks	200 μl of HUA solution (containing 200 mg/kg hypoxanthine and 30 mg/kg yeast extract) and 250 mg/kg potassium oxonate for 12 weeks	Bacteroidetes, Firmicutes and Patescibacteria↑; Actinobacteria and Proteobacteria↓	Reduce the serum UA by inhibiting UA biosynthesis and reabsorption; reduce the richness and diversity of the gut microbiota; involved in pluripotency of stem cell regulation, mTOR signaling pathway, and proteoglycans	(Fan et al., 2022)
	Apostichopus japonicus Oligopeptide (AJOP)	50 mg/kg AJOP by gavage for 8 weeks	A mouse model of HUA induced by purine-rich solution (containing 200 mg/kg hypoxanthine and 30 mg/kg yeast extract) and 250 mg/kg potassium oxonate in combination by oral gavage for 8 weeks	Coriobacteriaceae, Ruminococcaceae, Bacteroidaceae, and Helicobacteraceae↑	Regulate UA metabolism and alleviate renal inflammation; alter intestinal integrity, SCFAs production, and m6A methylation; alter gastrointestinal tract microbiota profiles	(Lu et al., 2021)
	The enzymatic hydrolysates of *Apostichopus japonicus* (EH-JAP) and *A. leucoprocta* (EH-LEU)	150 mg/kg EH-JAP and 150 mg/kg EH-LEU, respectively, by gavage daily for 8 weeks	A mouse model of HUA induced by 200 μl of high urine solution (200 mg/kg hypoxanthine, 30 mg/kg yeast extract, and 200 mg/kg potassium oxalate)	*Lactobacillus* and SCFAs producers↑; Opportunistic pathogens↓	Downregulate the transcription of pro-inflammatory cytokines; upregulate the transcription of anti-inflammatory cytokines; inhibit the activation of the TLR4/MyD88/NF-κB signaling pathway; alleviate the dysfunction of the gut microbiota	(Wan et al., 2020)
Others	Fisetin	Fisetin (50 and 100 mg/kg) was given by gavage and dissolved in 20% PEG 400	A mixture of adenine (160 mg/kg) and potassium oxonate (2,400 mg/kg) was administrated every other day for 4 weeks to induce HUA-induced chronic kidney disease	Bacteroidetes and Epsilonbacteraeota↑; Firmicutes↓	Lower serum UA and improve kidney injury; modify the structure and composition of gut microbiota; regulate plasma amino acid metabolism, and modulate gut microbiota-derived L-Tryptophan metabolism; alleviate kidney fibrosis by inhibiting L-kynurenine-induced aryl hydrocarbon receptor activation	(Ren et al., 2021)
	Nuciferine	Oral administration of nuciferine (25 mg/kg) 1 h later after potassium oxonate	A rat model of HUA induced by potassium oxonate (250 mg/kg)	*Lactobacillus* and *Enterococcus*↑; *Escherichia-Shigella* and *Bacteroides*↓	Differential metabolites interact closely with Firmicutes and Bacteroidetes	(Wang et al., 2020)

↓, decrease the relative abundances; ↑, increase the relative abundances.

#### 4.2.1 Anti-gout drugs

In addition to lifestyle improvements, drug therapy is an important approach in the treatment of HUA and gout and includes allopurinol and febuxostat, which inhibit UA production; benzbromarone, which promotes uricosuric excretion; and febuxostat, a xanthine oxidoreductase inhibitor. Recent studies have shown that in addition to improving UA symptoms, these drugs have positive effects in regulating the gut microbiota. A restriction of gut microbiota biodiversity was detected in untreated gout patients, and febuxostat partially restored this alteration (Lin et al., 2021). Biochemical metabolic indices involved in liver and kidney metabolism were significantly correlated with the composition of the gut microbiota in patients with gout. After the treatment of HUA with allopurinol and benzbromarone, the intestinal microbiota of rats changed. Both drugs could lead to an increase in *Bifidobacterium* and *Collinsella* and a decrease in *Adlercreutzia* and *Anaerostipes* after the reduction in UA (Lin et al., 2021). In addition, *Bilophila*, *Morganella*, and *Desulfovibrio* decreased after allopurinol treatment, while *Butyricimonas* and *Ruminococcus* decreased and *Proteus* increased after benzbromarone treatment (Lin et al., 2021). The above studies suggest that anti-gout drugs may exert a UA-lowering effect by regulating the gut microbiota, but their correlation needs to be further studied.

#### 4.2.2 TCM formulas

Although the effect of Western medicine in the treatment of gout is currently obvious, there are problems, such as easy recurrence and some adverse reactions after drug withdrawal. Traditional Chinese medicines (TCMs) have a long history in the treatment of gout and have the advantages of multichannel, multitarget, and multilevel symptomatic treatment. TCMs exert therapeutic effects by lowering UA, exerting anti-inflammatory, antioxidation effects, and protecting the kidneys. CoTOL (consisting of *Glabrous Greenbrier Rhizome*, *Dioscorea septemloba Thunb*, *Curcuma Longa*, *Parasitic loranthus*, *Herba Siegesbeckiae*, *Maydis stigma*, *Semen Coicis*, and *Corydalis Rhizoma*) is a TCM formulation used clinically for the treatment of gout and HUA. An analysis of CoTOL targeted bacteria in a mouse model of obesity HUA inoculated with the XDH-producing bacterium *Streptococcus faecalis.* The results showed that the *S. faecalis* inoculation resulted in elevated UA and altered the gut microbiota structure, while the CoTOL treatment increased the abundance of *Akkermansia* and decreased the abundance of *Bacteroides* and *Alloprevotella (* Gao et al., 2020). In gouty arthritis model mice, Simiao Decoction can effectively reduce the serum UA levels, reduce myeloperoxidase (MPO), XOD, and adenosine deaminase (ADA) activities, and relieve gout-related symptoms such as foot swelling and pain (Lin et al., 2020). In addition, Simiao Decoction reduced some serum proinflammatory cytokines, including IL-1β, IL-9, IFN (interferon)-γ, macrophage inflammatory protein (MIP)-1α, and MIP-1β (Lin et al., 2020). By reducing potential pathogens and restoring gut microbiota, the gut ecosystem may be a potential anti-inflammatory target of Simiao Decoction. The serum UA levels were significantly decreased, and the fecal UA levels were significantly increased after an intervention with a TCM chicory (*Cichorium intybus* L.) (Bian et al., 2020). In addition, chicory can repair intestinal mucosal damage and improve the permeability of the intestinal barrier. A sequencing analysis showed that chicory restores the gut microbiota and alleviates HUA by increasing probiotic flora (*Bifidobacterium*, Erysipelotrichaceae) and reducing pathogenic flora (Helicobacteraceae) (Bian et al., 2020). Further studies showed that this may be related to the stimulation of intestinal uric acid excretion by regulating the expression of ABCG2 (Wang et al., 2017). The gut microbiota has become a new way to understand TCM and has great potential in the exploration of TCM waste, add-on therapy and TCM individualized precision medicine (Yue et al., 2019). In particular, the role of traditional Chinese medicine in regulating the gut microbiota should be considered to deepen our understanding of the functions of gut microbiota metabolites and the mechanisms of specific diseases (Cheng et al., 2021; Liu et al., 2022). Studying the effects of interventions with TCM formulas on the gut microbiota is beneficial for explaining the multichannel and multitarget characteristics acting on the body, thereby contributing to giving full play to the advantages of TCMs in fighting gout.

#### 4.2.3 Polysaccharides

Plant polysaccharides can improve the intestinal tissue morphology, maintain the integrity of the intestinal barrier, enhance the immune response, and regulate the gut microbiota, such that the internal environment of the intestinal tract is in a good state (Lovegrove et al., 2017). Polysaccharides can act as a unique carbon source for specific gut microbiota during fermentation and may be an active component in regulating the gut microbiota. A polysaccharide from *Ulva lactuca* (ULP) exerts a UA-lowering effect by regulating the gut microbiota, which is characterized by increasing the abundance of beneficial microorganisms while reducing the abundance of harmful bacteria to restore the homeostasis of the gut microbiota (Li et al., 2021). A polysaccharide from *Enteromorpha prolifera* (EPP) significantly reduced serum UA, BUN, serum XOD, and hepatic XOD in HUA mice. In addition, EPP maintained the stability of the gut microbiota, confirming that *Parasutterella* is closely related to the regulation of HUA (Li et al., 2021).

In recent years, studies have shown that dietary fiber, as one of the seven essential nutrients for the human body, can play an important role in immunity and metabolism by regulating the structure of the gut microbiota (Holscher, 2017; Gill et al., 2021). Dietary fiber cannot be digested and absorbed by the gastrointestinal tract and cannot produce energy. Adding dietary fiber to the diet can significantly change the diversity of gut microbes and increase the abundance of SCFA-producing flora (So et al., 2018; Zhao and Zhang, 2018; Dalile et al., 2019). Inulin supplementation can effectively relieve HUA, increase the expression of ABCG2 in the intestine, and downregulate the expression and activity of XOD in the liver of Uox-knockout mice (Guo et al., 2021). Further studies showed that inulin supplementation enhanced the microbial diversity and increased the relative abundance of beneficial bacteria, including SCFA-producing bacteria, such as *Akkermansia* and *Ruminococcus*. Furthermore, inulin treatment increased the production of SCFAs (acetate, propionate, and butyrate concentrations) derived from the gut microbiota in Uox-knockout mice (Guo et al., 2021), which was positively correlated with efficacy in relieving HUA. Adding dietary fiber-rich β-carotene and green tea powder to the diet reduced joint swelling and pain in mice with gouty arthritis; decreased serum UA and three proinflammatory cytokines, namely, IL-1β, IL-6, and tumor necrosis factor (TNF-α); improved the gut microbiota profile; and decreased purine and pyrimidine metabolism (Feng et al., 2022).

#### 4.2.4 Phenols

Phenolic compounds are widely present in plants and are secondary metabolites synthesized by plants during normal development. Phenols are not only a type of natural antioxidant, but also a type of important biologically active substance in food (Veiga and Costa, 2020). *Camellia japonica* bee pollen polyphenols changed the gut microbiota structure of HUA model mice and increased the abundance of beneficial bacteria such as *Lactobacillus*, and the content of SCFAs was also increased accordingly (Xu et al., 2021). Chlorogenic acid (CGA) reduces the UA, BUN, CRE, aspartate transaminase (AST), and alanine transaminase (ALT) levels and inhibits XOD activity (Zhou et al., 2021). CGA inhibited the activation of the TLR4/MyD88/NF-κB signaling pathway in the kidney, thereby reducing inflammation in HUA mice. Furthermore, CGA treatment increased fecal SCFA production and increased the relative abundance of SCFA-producing bacteria, including Bacteroidetes, Prevotaceae UGC-001, and Butyricimonas, and reversed purine and glutamate metabolism in the gut microbiota (Zhou et al., 2021).

#### 4.2.5 Peptides

Bioactive peptides have a positive effect on the life activities of living organisms. In recent years, such peptides have gradually become one of the research hotspots in the fields of food, health food, and special medical food. Studies have shown that proteases produced by microorganisms in the gut act on proteins in food to produce bioactive peptides. Furthermore, bioactive peptides have obvious regulatory effects on the structure of the gut microbiota, and changes in the structure of the gut microbiota have an important impact on the health of the host. Tuna meat oligopeptide (TMOP) attenuates HUA and renal inflammatory phenotypes, reprograms uric acid metabolic pathways, inhibits the activation of NLRP3 inflammasome and TLR4/MyD88/NF-κB signaling pathway, and inhibits p65-NF-κB phosphorylation (Han et al., 2020). Furthermore, TMOP treatment repaired the intestinal epithelial barrier, reversed gut dysbiosis, and increased SCFA production (Han et al., 2020). In a HUA mouse model, hexapeptides (GPAGPR and GPSGRP) reduced serum UA by inhibiting UA biosynthesis and reabsorption, and attenuated renal inflammation by inhibiting NLRP3 inflammasome activation. Both peptides, as potential microbiota modulators, reduced gut microbiota richness and diversity, altering the composition at the phylum and genus levels (Fan et al., 2022). *Apostichopus japonicus* oligopeptide (AJOP) can significantly alleviate HUA, regulate UA metabolism, inhibit the activation of NLRP3 inflammasome and NF-kB-related signaling pathways, and restore the m6A methylation levels (Lu et al., 2021). Furthermore, fecal microbiota transplantation (FMT) effectively alleviated HUA in mice by selectively modulating the corresponding pathways associated with AJOP treatment (Lu et al., 2021), suggesting that the underlying mechanism of AJOP’s protective effect is partially dependent on the microbiota. In a mouse model of diet-induced HUA, both EH-JAP and EH-LEU inhibited UA biosynthesis and promoted UA excretion (Wan et al., 2020), thereby alleviating the hyperuricemic phenotype. Furthermore, EH-JAP and EH-LEU treatments alleviate gut microbiota dysfunction by increasing the abundance of beneficial lactobacilli and SCFA producers and reducing the abundance of opportunistic pathogens (Wan et al., 2020).

#### 4.2.6 Other prebiotics

In HUA-induced chronic kidney disease (CKD), the flavonoid component fisetin improves renal function, renal fibrosis, and intestinal dysbiosis (Ren et al., 2021). The alkaloid component nuciferine can significantly relieve HUA. In addition, a 16S rRNA analysis revealed that diverse gut microbes were closely associated with changes in differential metabolites, especially bacteria from the phyla Firmicutes and Bacteroidetes, and suggested that urinary indoxyl sulfate and N-acetylglutamate may be potential biomarkers in addition to UA for the early diagnosis and prevention of HUA (Wang et al., 2020). Various components can play a prebiotic role by regulating the gut microbiota, protecting the intestinal mucosal barrier, and increasing the content of SCFAs, maintaining human health, or helping treat diseases. In the future, the development and utilization of related prebiotics should be strengthened.

### 4.3 FMT

FMT is a treatment method that transplants the intestinal microbiota of healthy hosts into patients through the digestive tract to restore the diversity of intestinal microbes. It is a current clinical research hotspot. The serum UA concentration is closely related to the balance of the intestinal microecology. Using fecal microbial transplantation to maintain intestinal microecology in patients with HUA and gout may be a new treatment method. Washing microbiota transplantation reduces the serum UA levels in gout patients, is associated with a reduction in the frequency and duration of acute gout flares, reduces the diamine oxidase and endotoxin levels, and helps improve their damaged gut barrier function (Xie et al., 2021). The antihyperuricemic effects of TMOP and AJOP can be transmitted by transplanting fecal microbiota from TMOP/AJOP-treated mice (Han et al., 2020; Lu et al., 2021). Anserine treatment reversed gut dysbiosis, repaired the intestinal epithelial barrier, and increased SCFA production, and an anti-HUA effect was demonstrated by the transplantation of fecal microbiota from anserine-treated mice (Han et al., 2021). Although the current evidence concerning the beneficial effects of fecal microbial transplantation on human health is insufficient, fecal microbial transplantation currently provides more treatment modes for patients with HUA and gout and has important implications for human health.

## 5. Discussion

Intestinal dysbiosis in patients with gout mainly manifests as an increase in opportunistic pathogens and a decrease in bacteria that promote the production of anti-inflammatory cytokines. The gut microbiota is involved in the metabolism of purines and UA. An imbalance in the gut microbiota could increase the concentration of UA, and the long-term deposition of UA in the joints can cause gout. Intestinal dysbiosis may lead to the occurrence and development of gout by inducing the body to produce endotoxin, triggering chronic inflammation, or altering the metabolism of SCFAs. Thus far, some progress has been achieved in research investigating the relationship between the gut microbiota and HUA and gout (Chu et al., 2021; Yin et al., 2022), which not only helps elucidate its pathogenesis but also may provide a new direction for the diagnosis and treatment of HUA and gout, which may provide a new target for the treatment of gout.

In the prevention and treatment of HUA and gout, maintaining intestinal microecological balance plays an important role in the regulation of UA, which may provide new hope for the treatment of gout patients. At present, through microecological therapy, such as probiotics, prebiotics, and fecal microbial transplantation, adjusting the intestinal microecological balance to prevent HUA and gout is a clinical research hotspot. At the same time, however, many questions remain to be resolved. On the one hand, it is easy to breed other microorganisms during the microbial culture. Therefore, it is very important to ensure the safety of microorganisms and prevent contamination by miscellaneous bacteria. In addition, there are differences in UA and purine metabolism between humans and experimental animals. Therefore, more clinical trials are needed to elucidate the mechanism of gut microbiota in HUA and gout patients. Another problem is that a patient’s condition needs to be considered, and an accurate diagnosis and proper examination are necessary. The use of gut microbiota in HUA and gout treatment is still in its infancy, and its efficacy and safety *in vitro*, *in vivo*, and in clinical trials need to be gradually verified before widespread application. The complexity of the gut microbiota, the lack of evidence-based medicine for new treatment methods, and the lack of clinical data all require further exploration in future research.

Currently, research investigating the mechanism of gut microecology leading to gout mainly focuses on biochemical aspects, such as UA metabolism and the release of inflammatory factors. High levels of UA and joint pain are caused by problems with the body’s immune response (Chen et al., 2017; Cabău et al., 2020; Joosten, 2020). Therefore, it is reasonable to believe that the intestinal microecology is closely related to the occurrence of gout immune mechanisms. Accelerating research investigating the relationship between gut microbiota and gout immunity could help elucidate the mechanism of gout. Furthermore, attention should be given to dynamic research concerning the gut microecology in gout to study the dynamic development process of the microecology in different stages and different living environments in gout and identify the key microorganisms and their functional genes related to the occurrence and development of gout. In addition, attention should be given to the influence of age, course of disease, symptoms, and other factors on the intestinal microecology in gout and the process and method of reconstruction after the destruction of the intestinal microecology in gout. On this basis, we focused on translational medical research developing gout gut microecological regulators, and standardized treatment methods.

## Author contributions

Conceptualization, ZW, JT; methodology, YL, JH, YL; software, WL, ZL; validation, ZW, JT; investigation, XX, YL; resources, ZB; data curation, WL; writing—original draft preparation, ZW; writing—revised manuscript, ZW, YL, WL; writing—review and editing, JT and JH; visualization, ZB; supervision, XX; project administration, ZW; funding acquisition, ZW and JT All authors have read and agreed to the published version of the manuscript.

## Funding

This work has been supported by Macao Science and Technology Development Fund (SKL-QRCM(UM)), Key R&D project of Sichuan Province (2022YFG0145), “Xinglin Scholar” Scientific Research Promotion Plan of Chengdu University of Traditional Chinese Medicine (BSH2021017).

## Conflict of interest

The authors declare that the research was conducted in the absence of any commercial or financial relationships that could be construed as a potential conflict of interest.

## Publisher’s note

All claims expressed in this article are solely those of the authors and do not necessarily represent those of their affiliated organizations, or those of the publisher, the editors and the reviewers. Any product that may be evaluated in this article, or claim that may be made by its manufacturer, is not guaranteed or endorsed by the publisher.
